# A Large Size Chimeric Highly Immunogenic Peptide Presents Multistage *Plasmodium* Antigens as a Vaccine Candidate System against Malaria

**DOI:** 10.3390/molecules22111837

**Published:** 2017-11-01

**Authors:** José Manuel Lozano, Yahson Varela, Yolanda Silva, Karen Ardila, Martha Forero, Laura Guasca, Yuly Guerrero, Adriana Bermudez, Patricia Alba, Magnolia Vanegas, Manuel Elkin Patarroyo

**Affiliations:** 1 Department of Pharmacy, Universidad Nacional de Colombia, Bogotá DC 111321, Colombia; yfvarelaq@unal.edu.co (Y.V.); kjardilap@unal.edu.co (K.A.); lkguascap@unal.edu.co (L.G.); miakasan@gmail.com (Y.G.); mepartarr@gmail.com (M.E.P.); 2 Fundación Instituto de Inmunología de Colombia (FIDIC)-Universidad del Rosario, Bogotá DC 111221, Colombia; yolisica@gmail.com (Y.S.); mforerogar2000@gmail.com (M.F.); adriana.bermudez@urosario.edu.co (A.B.); mpalbas@unal.edu.co (P.A.); magnolia.vanegas@urosario.edu.co (M.V.)

**Keywords:** *Plasmodium*, chimeric immunogen, macromolecule synthesis, malaria, vaccine candidate

## Abstract

Rational strategies for obtaining malaria vaccine candidates should include not only a proper selection of target antigens for antibody stimulation, but also a versatile molecular design based on ordering the right pieces from the complex pathogen molecular puzzle towards more active and functional immunogens. Classical *Plasmodium falciparum* antigens regarded as vaccine candidates have been selected as model targets in this study. Among all possibilities we have chosen epitopes of *Pf*CSP, STARP; MSA1 and *Pf*155/RESA from pre- and erythrocyte stages respectively for designing a large 82-residue chimeric immunogen. A number of options aimed at diminishing steric hindrance for synthetic procedures were assessed based on standard Fmoc chemistry such as building block orthogonal ligation; pseudo-proline and microwave-assisted procedures, therefore the large-chimeric target was produced, characterized and immunologically tested. Antigenicity and functional in vivo efficacy tests of the large-chimera formulations administered alone or as antigen mixtures have proven the stimulation of high antibody titers, showing strong correlation with protection and parasite clearance of vaccinated BALB/c mice after being lethally challenged with both *P. berghei*-ANKA and *P. yoelii* 17XL malaria strains. Besides, 3D structure features shown by the large-chimera encouraged as to propose using these rational designed large synthetic molecules as reliable vaccine candidate-presenting systems.

## 1. Introduction

Malaria, a devastating disease caused by *Plasmodium* parasites; is transmitted to humans by the bite of a female *Anopheles* mosquito. In spite of efforts, from the five parasites that infect humans, *Plasmodium falciparum* is the most lethal, being responsibly for 429,000 deaths in 2016 [1]. An effective malaria vaccine remains as a hope for the disease prevention and morbidity/mortality reduction.

Pre-erythrocyte and blood parasite stages exhibit important antigens that have been regarded as targets for a malaria vaccine. Currently the *RTS,S* recombinant vaccine which is composed by antigens from hepatitis B and the *P. falciparum* circumsporozoite (*Pf*CSP), has a moderate effectiveness which decreases with vaccination time [2].

A total protective immunity by an “ideal vaccine” against malaria appears to be an *utopia* that is why most parasite life-cycle stages have to be considered when developing more potent vaccines. Hence improving molecular designing of novel antigens regarding all potential key-point strategies used by *Plasmodium* spp. to evade the host immune mechanisms, as well as ensuring a successful antigen presentation and to resemble those specific highly regulated molecular 3D arrangements of pathogen ligands have to be considered.

Studies conducted in Colombia have aimed to design candidates based on multi-stage components. Firstly, the synthetic SPf66 vaccine showed a moderate protective efficacy for which it was not followed further studies [3,4]. Recent works have proposed an approach to develop malaria vaccine candidates based on identification of peptides from the parasite conserved protein regions, which specifically bind to host cells. Once identified these active peptides are modified by replacing their critical binding residues to produce synthetic peptide analogs [5].

Synthetic procedures employ solid supports where the peptide chains are anchored; and the growing peptide is obtained by systematic coupling of desired *N*-α-protected amino-acids. However standard procedures are limited to produce short peptides and do not achieve a 100% peptide chain completeness mainly due to steric hindrance, thus inducing “difficult sequences” resulting in low yields and limited purity [6].

To overcome such problems, the use of polystyrene, acrylamide and polyethylene based solid supports have been proposed as well as chaotropic agents and microwave-assistance strategies [7]. Hence in order to optimize the synthesis process of a large peptide sequence evidencing potential synthetic difficulties, it would be first necessary to define a rational strategy to improve its production yield and purity.

This study was aimed to develop an 82-mere chimeric peptide based on four highly immunogenic *Plasmodium falciparum* merozoite and sporozoite surface antigens. Therefore a large chimera construct was envisioned by strategically positioning those four selected conserved antigenic peptides which contained specific amino acids replacements in their high activity binding motifs to lead a large chimeric immunogen. Importantly, peptide sequences from *Plasmodium falciparum* sporozoite and merozoite antigens have been identified as critical for parasite invasion. Among a number of possibilities of selecting *Plasmodium* targets to be considered in this work we have chosen four representative antigens which have been regarded each as a potential malaria vaccine candidate. As mentioned, high activity binding peptides were selected from the *P. falciparum* circumsporozoite protein (*Pf*CSP), the sporozoite threonine-asparagine-rich protein (STARP), the merozoite surface antigen-1 (MSA1) and the ring infected surface antigen (*Pf*155/RESA) [8,9,10,11]. The next challenge in our design consisted in defining each modified antigen position into the 82-mere large chimera. To solve such a puzzle many combinations of those four components were tested regarding potential synthetic hindrance, one of them emerged as a reliable. Therefore an experimental design led to propose a specific order and location of these four components into a 82-mere chimeric peptide which was defined as *N*-*Pf*CSP^68–87^-STARP^24–43^-*Pf*155/RESA^141–160^-MSA1^1283–1301^-*C*; and was then obtained by Fmoc chemistry with and without microwave-assistance and was subjected to physicochemical and structure characterization. Besides monomer forms of each modified antigen, polymer versions thereof were proposed in order to be simultaneously tested for their immunogenic as well as for their functional anti-*Plasmodial* activity in parallel assays. Thus for obtaining such polymer versions of each antigen, Cys residues were positioned on each antigen’s *N*- and *C*-termini. In order to test the chimeric peptide protecting capacity against malaria, in vivo tests on BALB/c mice experimentally challenged with two different rodent malaria species (*Plasmodium berghei ANKA* and *Plasmodium yoelii 17XL*) were conducted.

## 2. Results and Discussion

### 2.1. Plasmodium Falciparum Protein and Peptide Targets

**Figure 1:**
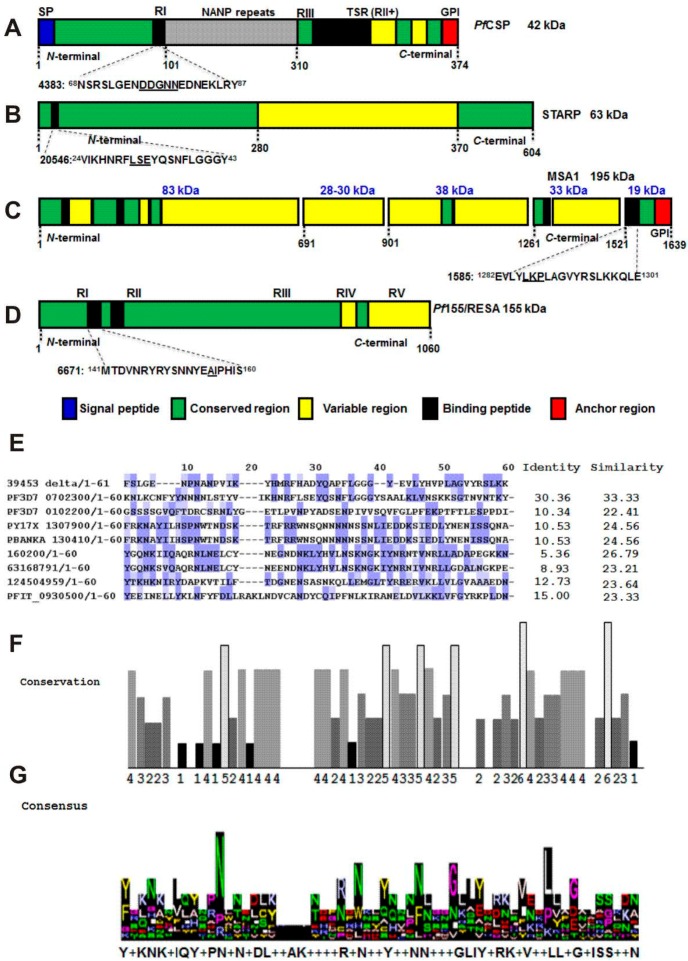
Genetic organization of *P. falciparum* antigen-peptide targets and bioinformatics. (**A**) *Pf*CSP sporozoite protein and its 4383 peptide; (**B**) STARP sporozoite antigen and its native 20546 peptide; (**C**) MSA1 merozoite protein and its 1585 native peptide; (**D**) *Pf*RESA merozoite antigen and its 6671 native peptide; (**E**) Alignment of the large-chimera edited sequence displaying only the *Plasmodium*-derived sequences (39453_delta chimera). Color code for sequence identify: 80% mild blue; higher than 60% in light blue; higher than 40% in light gray and lower than 40% in white; (**F**) Sequence conservation by Jalview-EBI where the 11* score represents identical, 8 and 7 conserved, and 6 and 5 semi-conserved amino-acids respectively; (**G**) A logo-fashion for a consensus sequence is shown in different colors for amino-acids.

The amino-acid sequences of *Pf*CSP, STARP, *Pf*155/RESA and MSA1 were downloaded from the GenBank. *P. falciparum* 3D7 *Pf*CSP (CAB38998.2) and *P. falciparum* NF54, (AAA29527.1); *P. falciparum* STARP (CAA81224); *P. falciparum* FC27 *Pf*155/RESA, (P13831.1) and *P. falciparum* Wellcome MSA1 (CAA26676.1) from UniProtKB/Swiss-Prot. *Plasmodium* target proteins genetic-block structure can be observed in Figure 1A–D; and their selected native peptides belong to low-polymorphic regions. Critical amino-acids for parasite binding (underlined) were substituted as proposed [5]. The whole family of molecules physicochemical characteristics can be observed in Table 1.

### 2.2. Molecular Design, Synthesis and Physicochemical Characterization of the 82-Residue Chimeric Immunogen

Peptide surrogates were strategically positioned into a rationally-designed large chimera (39543) and spaced each other by Gly-Gly amino-acid pairs and amino-acid substitutions are in bold (Table 1). Molecular design proposes a chimeric immunogen considering its lowest synthetic hindrance and its highest immunogenic potential as a single large molecule. Four 20-amino-acid length surrogates representing each component were obtained in standard yields of 60% to 70% on 0.45 meq/g substituted resins, while the large 82-amino-acid length chimeric peptide obtained by either a standard method or by microwave-assisted radiation had 9.16% and 12.67% yields on 0.20 meq/g substituted resins respectively. Further experiments for obtaining the large-chimera produced 15.50%, 11.32% and 12.24% yields on 0.15 meq/g, 0.10 meq/g and 0.08 meq/g resin substitutions. Ongoing experiments applying different synthetic strategies for obtaining the 82-mere large chimera such as ligation-condensation of large peptide blocks are being conducted in our group in order to improve overall synthetic yields. Molecular purity, identity and secondary structure-element profiles for the chimera and its single components can be observed in Supplementary Material Figure S1.

### 2.3. Bioinformatic Studies of Malarial Peptide Targets

Gly-Gly amino acid pairs were introduced as spacers into the 82-mere chimera for synthetic purposes as well as to serve as processing target sequences on APCs as being one of the cathepsins’ substrates; however those pairs were excluded for bioinformatic analyses in order to avoid any sequence bias, and as such those were preserved for all functional and in vivo experiments. Therefore when comparing the 61 amino-acids 39453_delta chimera lacking Gly-Gly spacers, regarding its identified orthologous sequences of *P. falciparum* 3D7, *P. berghei* ANKA and *P. yoelii* 17XL, important homology percentages were estimated as judged by similarity and identity indexes (Figure 1E).

Identity scores ranged from 10.53% to 30.36% among the three *Plasmodium* sequences, while having similarity values from 23.21% to 33.33%, which are statistically significant. Structural homology, represented by highlighted residues (Figure 1E), can be associated directly to *Pf*CSP, STARP, *Pf*155/RESA and *Pf*MSA1 native amino-acid sequences. In spite that relevant amino-acid residues for protein binding were strategically mutated on the designed chimera, small score values reflect those changes; however there is still a high structure-conservation degree. As observed in Figure 1F, amino-acids belonging to the chimera template regarding specific *Plasmodium* species possess a high significant conservation degree, hence 51 out of 61 residues representing an 83.61%, hold high conservation scores (up to 6) as analyzed by JalView [12,13]. As seen in the logo representation generated by JalView shown in Figure 1G, a statistically significant consensus sequence is proposed Y+KNK+IQY+PN+N+DL++AK++++R+N++Y++NN+++GLIY+RK+V++LL+G+ISS++N.

### 2.4. Large Chimera Peptide Immunological Properties

**Figure 2:**
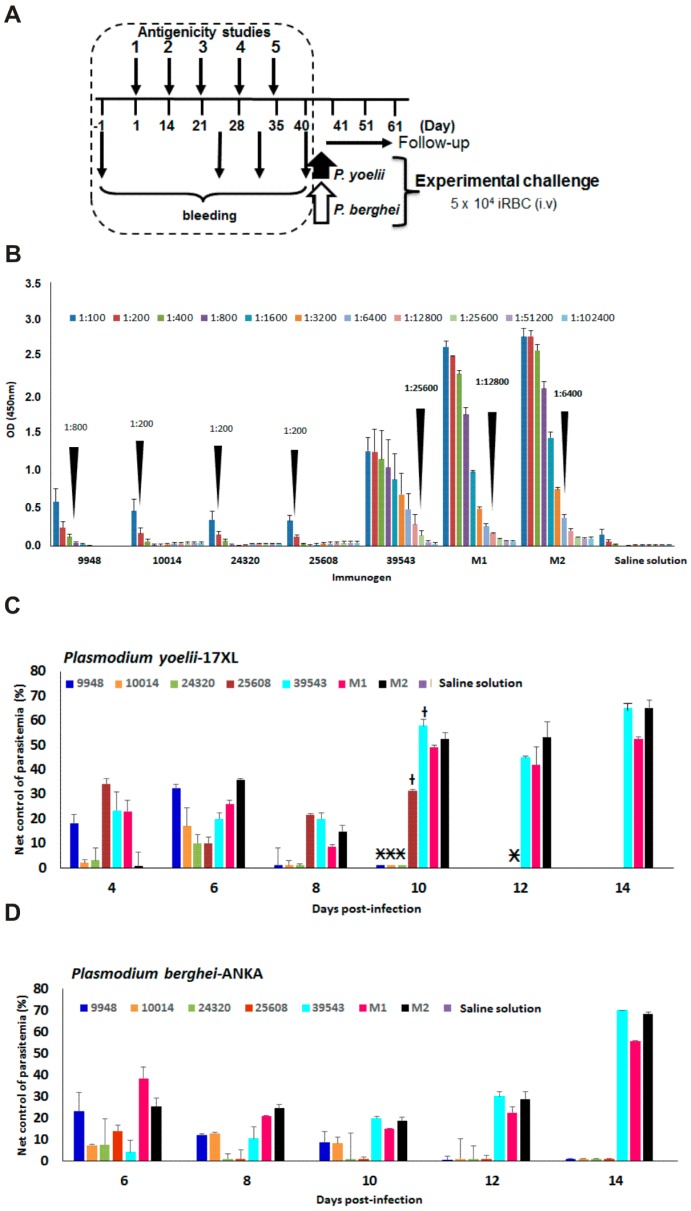
*P. falciparum*-based large chimera antigenicity and protection capacity. (**A**) A 5-dose immunization scheme consisted in a first dose and four boosts at days 14, 21, 28 and 35. Bleeding samples were obtained previously to the first vaccination and after the third, fourth and fifth dose. Malaria lethal challenging was conducted by i.v injection of 5 × 10^4^ *P. yoelii* and *P. berghei* infected RBCs and parasitemia follow-up for a 60-day period; (**B**) Immunogen-formulations were 9948-*Pf*RESA, 10014-MSA1, 2430-STARP, 25608-*Pf*CSP, 39543 large-chimera, a mixture of the four single components **M1**, a mixture of the four components in presence of the large-chimera **M2** and saline solution for the placebo group. Antibody titers were defined as the sera highest dilution that still yields a positive reading expressed as the OD mean value at 450 mn produced by a given analyte sample minus twice the OD mean value of pre-immune sera ± SD; (**C**) Net control of parasitaemia of vaccinated mice experimentally challenged with *P. yoelii 17XL* and with *P. berghei ANKA* in (**D**). * spontaneous death and † mild symptoms.

A five dose administration scheme led to stimulation of antibody titers in all immunized BALB/c mice (Figure 2A) which received polymeric forms of each single peptide component (groups 1 to 4 in Table 2), the large-chimera alone (group 5), a mixture of the four components **M1** (group 6), a mixture of the four components plus the large-chimera.

**M2** (group 7), and a placebo group administered with saline solution (group 8). As seen in Figure 2B, all single components represented by peptides coded 9948-*Pf*155/RESA, 10014-MSA1, 24320-STARP and 25608-*Pf*CSP induced low-medium antibody titers ranging from 1:200 to 1:800. Interestingly, the group of mice vaccinated with the mixture **M1** (four single components) stimulated 1:12,800 antibody titers. The group immunized with the mixture plus the large-chimera **M2** antibody titers of 1:6400 and remarkably the mice group vaccinated with the large-chimera alone induced the highest level of antibody titers being 1:25,600. The placebo group did not show any antibody reactivity. Interestingly the four-component mixture-formulation **M1** proved to be higher antibody inducer than a mixture **M2** composed by the four-components supplemented with the large-chimera.

**Figure 3:**
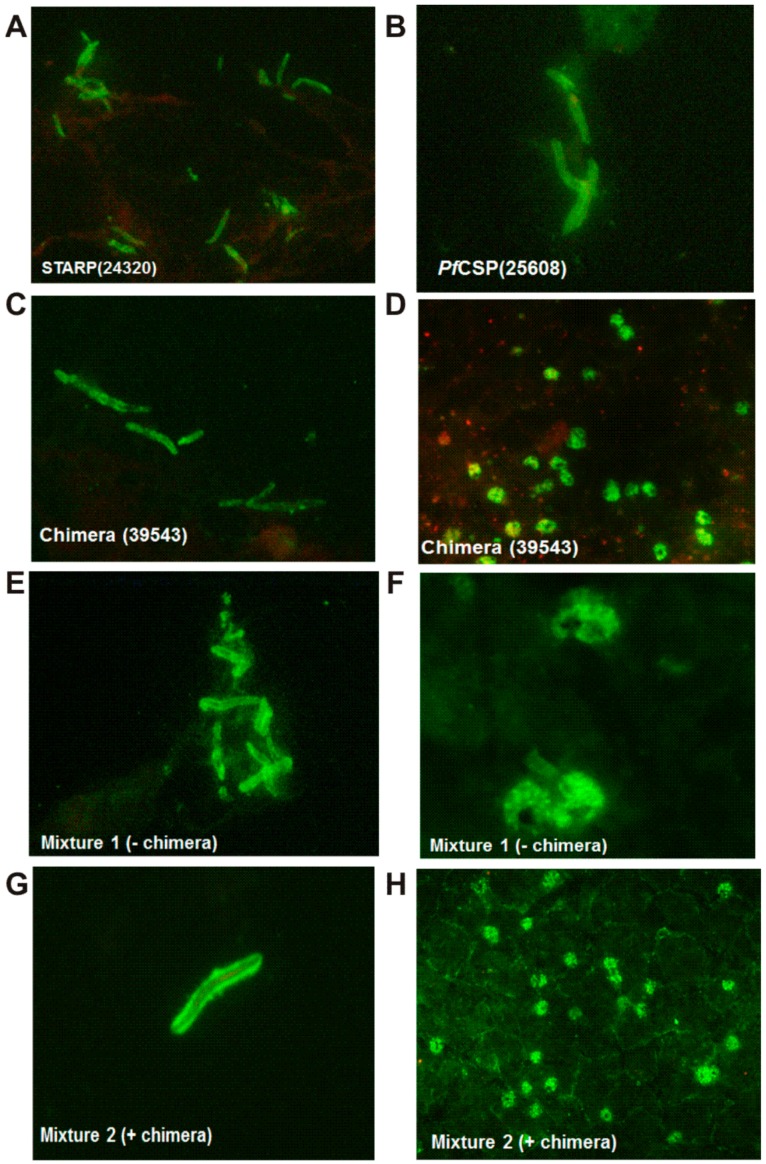
Vaccinated mice antibody reactivity to *P. falciparum* native antigens by IFA. *P. falciparum* sporozoite forms detected by antibodies induced by (**A**) 24320-STARP; (**B**) 25608-*Pf*CSP and (**C**) the large chimera; (**D**) *P. falciparum* mature schizonts revealed by antibodies to the large chimera; (**E**) Sporozoites detected by antibodies to the **M1** mixture; (**F**) Mature schizonts revealed by antibodies to the **M1** mixture; (**G**) Sporozoites recognized by antibodies to the **M2** mixture and (**H**) Mature schizonts recognized by antibodies to the **M2** mixture.

In addition IFA experiments led to demonstrate these antibodies specificity for a given *P. falciparum* stage. As shown in Figure 3A,B, antibodies to STARP and *Pf*CSP peptides strongly react with sporozoites and did not recognize *Plasmodium* merozoites. Interestingly antibodies stimulated by the large-chimera strongly cross-react with both sporozoites and merozoites (Figure 3C,D), while antibodies to *Pf*155/RESA and MSA1 only recognize merozoites (data not shown). Likewise antibodies form animals vaccinated with mixtures **M1** in absence (Figure 3E,F) or in presence of the large-chimera **M2** (Figure 3G,H), cross-react similarly with both sporozoite and merozoite stages of *Plasmodium falciparum*, as expected.

### 2.5. Functional In Vivo Activity of the Large Chimera on BALB/c Mice Infected with Rodent Malaria Strains

After antibody reactivity was proven each animal group was split and intravenously challenged with lethal doses of *P. yoelii* the first and *P. berghei* the second subset. As shown in Figure 2C,D, parasitemia control levels of tested mice revealed that the large-chimera alone and a mixture of its single components supplemented with the large-chimera (**M2**) stimulated functional neutralizing antibodies which harbor strong immunoreactivity against both rodent *Plasmodium* strains specially 10 days after challenge. As displayed in Figure 2, none of the four basic components 9948-*Pf*RESA, 10014-MSA1, 24320-STARP and 25608-*Pf*CSP formulations was able to stimulate a significant humoral response that could be associated in controlling the plasmodial infection; therefore most groups administered with basic components rapidly developed high parasitemia levels and died before the 10th day after challenged. On the contrary, antibodies to the large-chimera and antigen-mixtures in presence or absence thereof efficiently controlled and resolved the *Plasmodium yoelii* infection even after the 14th day after challenge (Figure 2C). The large-chimera showed an efficient immunological activity since when administered alone as a vaccine-formulation is able to induce neutralizing antibodies to the malaria infection.

Concerning the mice subgroups challenged with *Plasmodium berghei*, only animals vaccinated with antigen mixtures supplemented or not with the large-chimera, were able to stimulate an efficient humoral response associated to infection control and parasite clearance, being the formulation supplemented with the large-chimera (**M2**) and those vaccinated with the large chimera most actively resolved the plasmodial infection (Figure 2D). Interestingly the large-chimera alone had a remarkable effect on *P. berghei* parasitemia control in this subgroup of animals, in spite of the *P. berghei* higher virulence regarding the *P. yoelii* strain and outstandingly the chimera is required as an essential component of a mixture formulation (**M2**) and its role in protection and infection clearance became evident as observed in Figure 2D.

Thus, as evidenced in this work, a rationally designed large-chimera with the purpose of presenting specific antigens from different *Plasmodium* stage proteins, become an efficient molecular system for inducing a functional humoral response in vaccinated individuals, and so due to its potential as vaccine candidate, a number of experiments aimed to assess its processing and presenting mechanism have to be further performed.

### 2.6. Structure Properties of the Large Chimera Peptide

A high molecular weight chimeric-immunogen permitted us choosing four low-polymorphic peptides so coded 6671-*Pf*RESA^141–160^ and 1585-MSA1^1282–1301^ and 20546-STARP^24–43^ and 4383-*Pf*CSP^68–87^, which were then modified by selective specific amino-acid substitution (Table 1), and coded as 9948-*Pf*RESA, 10014-MSA1, 24320-STARP and 25608-*Pf*CSP respectively. This surrogate family served as the basis for generating a large-chimeric peptide (39543) whose basic structure and characteristics can be seen in Table 1.

**Figure 4:**
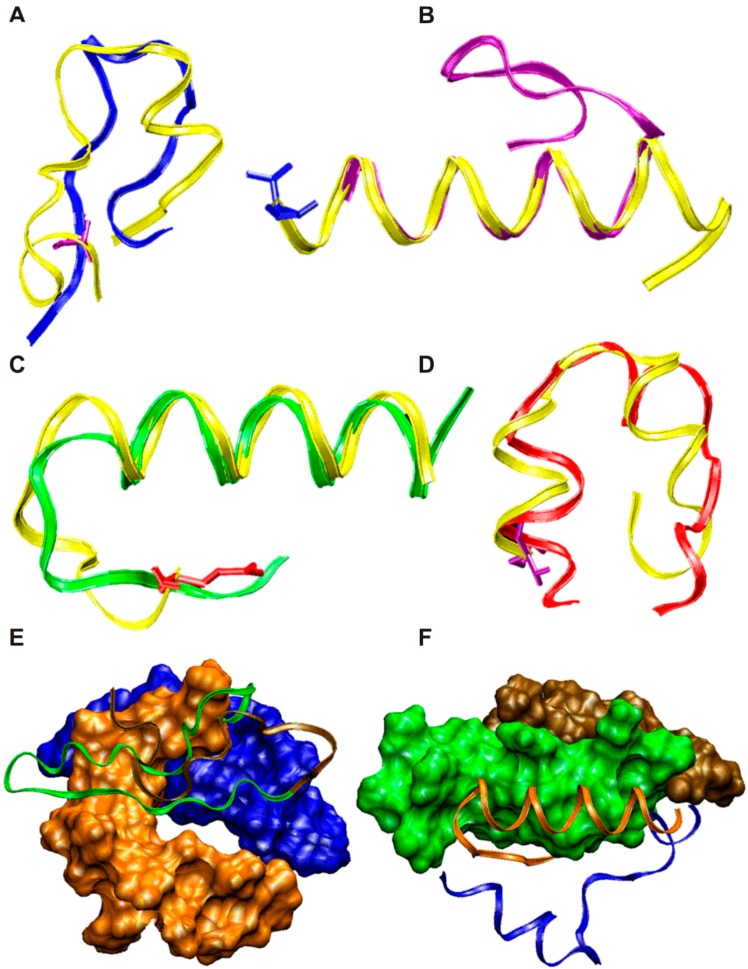
Structural properties of *P. falciparum* peptide targets and its large-chimera peptide. (**A**) 25607 modified *Pf*CSP^68–87^ (blue ribbon); (**B**) 24319 modified STARP^24–43^ (lilac ribbon); (**C**) 10013 modified MSA1^1282–1301^ (green ribbon); and (**D**) 9947 *Pf*155/RESA^141–160^ (red ribbon). In all cases native peptides are shown in yellow ribbons and different colors for their surrogates. The large chimera peptide 39543 most representative molecular model is displayed as both, solvent accessible surface and flat ribbons. Epitope fragments on the chimera are colorful identified as being *Pf*CSP ochre, STARP green, MSA1 orange and *Pf*155/RESA blue surface respectively as shown in (**E**,**F**).

Evidence obtained from experiments encouraged us to analyze this molecular family’s 3D conformational properties as an important step towards elucidation of immune molecular interactions. Thus, bi-dimensional ^1^H-NMR experiments provided database coordinates as Protein Data Bank *pdb* files for both the native as well as their surrogates, which served for molecular modelling. Molecules backbones are presented as ribbons, yellow for non-modified native sequences and other colors for modified surrogates (Figure 4A–D).

The sporozoite native *Pf*CSP^68–87^ fragment reveals β-strand-like elements and strongly differs to its modified-version (blue ribbons) which shows instead high-energy stretches as observed in Figure 4A. Similarly the native merozoite *Pf*155/RESA^141–160^ peptide discloses two α-helical stretches and strongly differs from its surrogate (red ribbon) which modulates such domains to randomly organized conformations (Figure 4D).

In addition, the native sporozoite-STARP^24–43^ peptide 3D structure profile shown in Figure 4C, highly overlap to its surrogate (green ribbon) in about a 90% of the 3D conformation and differing at their N-terminal portions. Likewise, the native merozoite MSA1^1282–1301^ fragment regarding its modified version (lilac ribbons), displayed similar 3D structure conformations revealing a high α-helix content and a low overlapping *C*-terminal portions (Figure 4B).

On the other hand, homology predictive 3D structure methods were employed for estimating and validating the large-chimera peptide conformation [13]. Thus, the 82-mere amino-acid sequence was submitted to the I-Tasser remote server (zhanglab.ccmb.med.umich.edu) for retrieving the most likely 3D models [14]. Data set was coded as S252625 for the submission. As a result a set of the top five most probably 3D models for this sequence were generated as coordinates’ data sets presented in *pdb* format. Following a validation quality process, the most probably 3D structure model was performed by submitting the previously obtained data set to the SwissModel remote server (swissmodel.expasy.org) and a Q-Mean index was also determined and used as a quality criteria [15]. The Q-Mean score is composed by a linear combination of 6 terms including energy, spatial and other contributing terms such as Z-score values regard high-resolution experimental structures of similar size solved by X-ray crystallography. Total Q-Mean-score for the most likely 3D model for the large chimera was 0.341 which is in the 0 to 1 range; and a −3.11 Z-score reflecting the estimated model reliability.

Visualization of validated 3D structure models was performed by using the generated-assessed 3D structure *pdb* files with the VMD (visual molecular dynamics) 1.8.6 software [16]. Figure 4E,F shows the 3D structure profile for the large-chimera 39543 revealing that the MSA1 and *Pf*155/RESA fragments, both from merozoite antigens, disclosed a high α-helix content (Figure 4F, orange and blue colored portions); meanwhile fragments derived from sporozoite antigens *Pf*CSP and STARP (ochre and green colors respectively) shown β-stranded structure features as seen in Figure 4E.

Therefore the proposed molecular arrangement for the large-chimerical peptide reveals intriguing 3D-structure features which could be associated to its functional immunogenic properties and justify antibody titers stimulation harboring neutralizing capacity of *Plasmodium* infection and parasite clearance. As discussed the large-chimera displays two highly compact alpha-helical regions which are molecularly restricted by an intra-catenary hydrogen-bond network. These alpha-helical domains comprise both merozoite epitopes derived from *Pf*155/RESA and MSA1 antigens. The other two more flexible structure portions revealed β-stranded and random-coiled stretches which probably confers more free energy to the whole molecule as relevant entropic contributions to the chimera. As a consequence of its 3D-conformational restriction, the designed large-chimeric vaccine candidate, is capable of stimulating antibodies that efficiently cross-react with both plasmodial stages.

All conducted experiments presented in this work are based on a careful experimental design conducted to obtain a large 82-mere chimeric immunogen. A first challenge in this pursuit consisted in strategical positioning of each selected antigen into the large chimeric immunogen, thus a number of immunological and synthetic considerations were taken into account among a number of possibilities. Information regarding each component low polymorphism in the human and mouse *Plasmodium* spp. genomes led selecting this animal model for the chimera functional evaluation under controlled conditions. Therefore, peptide antigens containing high activity binding motifs to target cells from the *Plasmodium falciparum* circumsporozoite (*Pf*CSP), the sporozoite threonine-asparagine-rich protein (STARP), the merozoite surface antigen-1 (MSA1) and the ring infected surface antigen (*Pf*155/RESA) proteins, were selected for this study, knowing that all of them have been regarded previously as malaria vaccine candidates, but independently each did not reach extensive field evaluation due to their limited protective efficacy in pre-clinical and clinical trials when conducted. However those termed high activity binding peptides exposed on the four selected antigenic proteins have evidenced overcoming those mentioned immunological profiles when submitted to specific amino acids replacements into binding motifs as above explained. This is the reason which encouraged us to choose such strategy for obtaining modified versions for each native antigen, which were then used as the components of the large chimera.

Remarkably the four human *Plasmodium* selected antigens from pre-erythrocyte and blood stages revealed orthologous sequences on malaria rodent species especially in both *P. berghei* ANKA and *P. yoelii* 17XL strains. Bearing in mind the whole set of considerations, a construct organized as *N*-*Pf*CSP^68–87^-STARP^24–43^-*Pf*155/RESA^141–160^-MSA1^1283–1301^-*C*; was proposed and synthesized.

The obtained data demonstrate that this large chimera construct was safe, antigenic and shown both immunological and functional profiles when tested in two controlled rodent model malarial experiments, thus the rationale behind the proposed experimental design seem to be in line towards obtaining novel multi-component malaria vaccine candidate systems, which have to be further evaluated by using a number of *Plasmodium* antigens and other animal experimental models closer to humans.

## 3. Materials and Methods

### 3.1. Plasmodium Falciparum Protein and Peptide Targets

*Plasmodium* peptide and protein sequences were analyzed and downloaded from the Gene Bank (http://www.ncbi.nlm.nih.gov) the Protein Data Bank (PDB, http://www.rcsb.org/pdb) as well as from the *Plasmodium* genome resource (http://plasmodb.org/plasmo/) was employed for obtaining protein orthologous proteins present in the *Plasmodium berghei* and *Plasmodium yoelii* genomes. As it is well known, *Plasmodium* circumsporozoite surface antigens constitute important targets for pre-erythrocyte vaccine development, thus *Pf*CSP has been extensively regarded as a potential vaccine candidate as elsewhere reported [17]. Further protein binding and 3D structure studies lead to identify a high activity binding peptide (HABP) coded 4383 on the *Pf*CSP structure [8]. Similarly STARP, a *Plasmodium* antigen is expressed on the surface of sporozoite forms and seem to play a key role in hepatic cells infection as described [18]. Subsequent cell specific binding and molecular structure tests lead identifying the so-coded 20546 peptide on the STARP primary structure as reported [9]. On the other hand merozoite targets for a malaria vaccine candidate have regarded the *Pf*155/RESA antigen, whose amino acid sequence was originally obtained by cloning experiments of a *P. falciparum* isolate FCQ27/PNG (FC27) (nucleotides 1-3268) as reported [19]. As above, in subsequent receptor-ligand interaction studies a high activity binding peptide (HABP) coded 6671 was identified from the *Pf*155/RESA primary structure [20]. Immunological and 3D structure properties were later reported [11]. Besides, a merozoite expressed antigen named MSA1 has been identified as one of the *Plasmodium* most abundant merozoite membrane antigens and so it has been regarded as an important target for neutralizing parasite erythrocyte-stages [21]. Therefore some HABPs were identified among the coded 1585 [22]. Immunological-3D structure studies allowed proposing the 1585 peptide as an important *Plasmodium* target for vaccine development [10].

### 3.2. Peptide Molecular Designing

Bearing in mind potential synthetic hindrance, as well as hypothetical immunological activity, a molecular design was proposed for the chimeric 82-mere peptide, which have included some Gly-Gly amino acid pairs as spacers of each peptide component of the chimera. Hence sequence organization for the substituted amino acid version of the template can be described as *N*-*Pf*CSP^68–87^-GlyGly-STARP^24–43^-GlyGly-*Pf*155/RESA^141–160^-GlyGly-MSA1^1283–1301^-GlyGly-*C*, that was coded as 39543 as can be observed in Table 1*.* Synthetic procedures included obtaining each single modified HABP component as being the coded 25607 the modified version of *Pf*CSP^74–78^, the 24319 peptide as the modified version of STARP^31–33^, the 10013 for MSA1^1286–1288^ and the 9947 being the modified version of *Pf*155/RESA^155–156^ respectively; Gly-Gly insertions were included in all cases. All possible permutations on the target sequence were submitted to a predictive synthesis hindrance by employing virtual tools such as the *Peptide Companion*^™^ software (Pharmaceuticals Inc., San Diego, CA, USA), thus the four peptide components were organized as having the lesser theoretical synthetic difficulty for a single large peptide design. Consequently an 82-residues chimeric linear peptide was submitted to solid phase synthesis procedures by Fmoc chemistry with or without microwave assistance. The so obtained target molecule was then subjected to physicochemical and immunological studies.

### 3.3. Solid Phase Synthesis of the 82-Residues Length Chimeric Peptide and Physicochemical Characterization

Rink-amide-ChemMatrix^®^-resin (PCAS BiomMatrix Inc., St-Jean-sur-Richelieu, QC, Canada), of 0.60 meq/g substitution was used as the solid support. Employed solvents were *N*,*N′*-dimethylformamide (DMF), 1-methyl-2-pirrolidone (NMP), dichloromethane (DCM) and isopropanol (IPA) were obtained from Sigma-Aldrich (St. Louis, MO, USA) and Panreac (Barcelona, Spain). Carbonyl function activating agents used were *N*,*N′*-dicyclohexylcarbodiimide (DCC), 1-hydroxybenzotriazol (HOBt), tetrafluoroborate-*o*-benzotriazol-yl-*N*,*N*,*N*,*N*-tetramethyluronium (TBTU) were obtained from AAPPTec (Louisville, KY, USA). Fmoc-*N*-α-amino acids were purchased from Merck (Heidelberg, Germany). Charged amino acid side-chains protecting groups were: 4-*tert*-butyl ester (OtBu) for aspartic acid (Asp, D) and glutamic acid (Glu, E), *tert*-butyl (*t*-Bu) for threonine (Thr, T), tyrosine (Tyr, Y) and serine (Ser, S), *tert*-butyloxycarbonyl (*t*-Boc) for lysine (Lys, K), trityl (Trt) for asparagine (Asn, N), glutamine (Gln, Q) and histidine (His, H), 2,2,4,6,7-pentamethyldihydrobenzofuran-5-sulfonyl (Pbf) for arginine (Arg, R). Reactor vessels were 20 mL polypropylene syringes adapted to polystyrene filters.

Synthesis of the 82-residue length chimera was performed from *C* to *N*-terminus; starting with the ChemMatrix Rink Amide resin (0.60 meq/g) swelling with 5 mL DCM and 5 mL NMP for 10 min. Resin substitution was adjusted to 0.20 meq/g by stoichiometrically coupling the first Fmoc-Gly-OH amino acid, which was previously dissolved in 5 mL NMP and activated with TBTU/HOBt/DIPEA (1:1:2, meq respectively), reaction was lead to proceed for one hour; acetylation was then conducted in order to cap any available primary amino function with 10 mL of an acetic anhydride/DMF/pyridine (1:1:1) solution for one hour, subsequent washes to the resin were carried out with DMF (2 times 1 min), IPA (3 times 1 min) and DCM (3 times 1 min). At this stage, Kaiser tests were performed to growing peptide-resin samples in order to verify capping completeness. Briefly, this test was conducted by mixing two drops of the A solution component of the ninhydrin reagent (40 g phenol/10 mL absolute ethanol, 1 mL of KCN stock solution (65 mg KCN/100 mL H_2_O and 50 mL pyridine) with one drop of the B component (1.25 g ninhydrin/25 mL absolute ethanol). After mixing and heating at 100 °C for 6 min, a blue-green color reveals a positive -NH_2_ content. On the contrary if the color appears yellow the test is considered as negative. For a quantitative approach for determining resin substitution degree after the first residue has been coupled, a few milligrams of peptide-resin were assessed by adding 0.5 mL of a piperidine/DMF (3:7) solution for 30 min, to which 6.5 mL methanol were then added and analyzed by spectrophotometrically reading at a 301 nm wave length [23].

RP-HPLC characterization of all peptides was performed in a Merck-Hitachi L-6200A chromatographic system (Merck, Tokyo, Japan) provided with a UV-VIS L-4250 detector which was adjusted to a 210 nm wave length. A semi-preparative Vydac RP-18 column of 250 mm × 4.6 mm and 5 μm particle size, was adjusted at room temperature for running samples at a rate flux of 1.0 mL/min for standard running samples.

Mass spectrometry analyses were conducted on a Bruker Daltonics Series Microflex MALDI-TOF spectrophotometer (Bruker Daltonics Inc., Billerica, MA, USA). A supersaturated α-cyano-4-hidroxycinamic acid (CCA) (Sigma Chemical Co., St. Louis, MO, USA). The CCA matrix was prepared as a saturated solution in 1 mL AT (40% Acetonitrile: 60% H_2_O- supplemented with 0.1% Trifluoroacetic acid). Samples were dissolved in TA to lead a 100 pmol/μL concentration. Samples were prepared for MALDI-TOF analysis by diluting the sample solution in the matrix-saturated solution, reaching a 10 pmol/μL concentration. Then, 0.5 μL aliquots of sample-matrix mixture were poured onto the target plate, air-dried and analyzed. Chimera and all peptide segments concentration was adjusted to 5.0 × 10^−4^ mg/μL for MS analysis; 2.5 μL of a solution containing a 2.5:18 dimer-matrix ratio was dispensed onto the analysis plate.

On the other hand Circular dichroism (CD) assays were carried out at room temperature on nitrogen-flushed cells using a Jasco J-810 spectropolarimeter (Jasco Co., Madrid, Spain). Spectra were recorded within a 190–260 nm wavelength interval, using a 1 mm path-length rectangular quartz cell. Each spectrum was obtained by averaging three scans taken at a 20 nm/min scan rate with 1 nm spectral bandwidth corrected for baseline deviation using Jasco software. The CD profile for each molecule was obtained by dissolving lyophilized purified peptides in water or 30% aqueous 2,2,2-trifluoroethanol (TFE), 0.5 mL final volume. A typical 0.2 mM peptide/peptide concentration in TFE–water mixture was stabilized but did not induce secondary structure in peptides, as described elsewhere [24].

### 3.4. NMR Studies, Structural Calculation, Peptide Prediction Structure and Molecular Visualization

Native peptides from *Pf*CSP^68–87^ 4383 and its modified 25608, STARP^24–43^ 20546 and its modified 24319, MSA1^1283–1301^ 1585 and its modified 10013 as well as *Pf*155/RESA^141–160^ 6671 and its modified 9947 respectively were analyzed and their structure elucidated. For NMR measurements, 10 mg of each lyophilized peptide, their analogues and chimera were dissolved in a H_2_O/D_2_O mixture (9:1 ratio), as well as in an aqueous 30% TFE solution (99.94% D 2,2,2-trifluoroethanol-d_3_ (Cambridge Isotope Laboratories, Andover, MA, USA) until reaching final 0.5 mL volume. Proton nuclear magnetic resonance (^1^H-NMR) spectra were recorded on a Bruker DRX 600 MHz spectrophotometer, provided with a temperature control unit. Spectra were recorded between 280 and 310 K using 4.5–5.0 pH range and referenced to water’s internal signal at 4.75 ppm. Routine COSYGSmtprtp (Avance version, Phase Sensitive) was performed to assign all spin systems, using gradient pulses for detection with multiple-quantum filter, according to gradient-radio experiments. TOCSY *mievgstp* 19 (Advance version, Homonuclear Hartman-Hahn Transfer), using mLEV 17 sequence mixing and 80–100 ms mixing-time, was carried out to corroborate NH and CαH side-chain connectivities for each spin system. Experiments (Advance version, 2D-Homonuclear Correlation via Dipolar Coupling Phase Sensitive using TPPI) were carried out for 150, 200, 300, 400 and 500-ms mixing-times to assign all sequential neighbors for the NOESY gstpi9-peptide chain. The 2D-data was processed on a Silicon Graphycs-Bruker Indy computer provided with XWINNMR 1.3 software (Bruker, Darmstadt, Germany). Temperature coefficients were determined from TOCSY spectra using a 285 to 315 K temperature range. A slope was deduced from a linear relationship established for chemical shift of temperature patterns for each hydrogen atom from amide groups (−ΔδHN/ΔT, ppm/K). 3JHNHα coupling constants were measured from the separation of multiplets in cross-peaks from unidimensional DFQ-COSY experiments.

The structural calculation was performed by using the software provided by Accelrys (Insight-II, Molecular Simulations Inc,, Accelrys Inc., San Diego, CA, USA) on Silicon Graphics work stations. NOE peaks, selected from 400 ms NOESY data sets, were integrated and converted into distance restraints. These restraints were grouped as strong, medium and weak corresponding to 1.8–2.5 Å, 2.5–3.5 Å, and 3.5–5.0 Å distance restraints respectively. Distance Geometry (DGII) software was used for producing 50 starting structures for each analyzed molecule, being in this case native and their modified analogues. These resulting 50 conformers were molecularly refined by using a strategy known as restricted molecular dynamic (rMD) as previously described [25].

Molecular modelling for all native antigens and their analogues was performed by adjusting distance ranges involving the likely hydrogen-bond NH...O were set at 1.8–2.5 Å. For molecular calculations all peptide bonds were forced to *trans* conformation and the Cα chirality to L. The distance geometry (DG) structures were refined using the restrained simulating annealing protocol (SA), which substantially reduces the force constraints at the beginning and selectively scales them up until the full values are regained. Distance-dependent dielectrics were used (4 × r). Procheck (www.ebi.ac.uk/thornton-srv/software/PROCHECK/) was employed to check the refined structures’ actual geometry with reasonable geometry. Thus, low constraint violations displaying a root mean square deviation (rmsd) values smaller than 2.7 were retained as it has been standardized in our work protocols in agreement to the literature.

Due to its poor solubility the native 6671 peptide (*Pf*155/RESA) was subjected to structure predictive analysis by using the PEP-FOLD 1.5 server, accessed from the URL http://bioserv.rpbs.univ-paris-diderot.fr/services/ in order to be compared with those 3D coordinates obtained by NMR experiments for its analogue coded 9948. Similarly, structure properties of the chimeric 82-residue peptide were analyzed by using the I-TASSER remote server accessed from the URL http://zhanglab.ccmb.med.umich.edu web site. The quality of both predicted molecular models was subsequently verified by using a remote server assistance which allowed by its structure assessment tools accessed from the URL http://swissmodel.expasy.org/.

### 3.5. Bioinformatic Studies

The chimera 82-residue length peptide sequence serves as the basis for a basic local alignment search tool (BLAST), was performed in the http://blast.ncbi.nlm.nih.gov/Blast.cgi server which lead finding regions of local similarity and sequence homology for *Plasmodium* antigenic proteins. As it was described above in experimental design, the a first input an edited sequence herein called 39453_delta chimera of 61 amino acids in length was employed, whose amino acid sequence is: −NSFSLGENPNANP−VIKYHMRFHADYQAPFLGGGY−EVLYHVPLAGVYRSLKKQLE−MTDVNRYRYSNNYEEEQHIS−. This sequence contains the modified peptide versions from *Pf*CSP, STARP, MSA1 and Pf155/RESA respectively. Thus a set of *Plasmodium falciparum* and *Plasmodium yoelii* proteins were identified. Then by using a second input consisting in a *P. yoelii* (PY17X_1307900) entry sequence lead identifying a *P. berghei* ANKA protein (PBANKA_1304100) which has a strong homology. Therefore such screening allowed us to obtain the followings orthologous sequences coded as 39453_delta, PF3D7_0702300, PF3D7_0102200 (*P. falciparum*), PY17X_1307900, (*P. yoelii*) PBANKA_130410 (*P. berghei* ANKA), gi|160200, gi|63168791, gi|124504959 and PFIT_0930500 (*P. falciparum*) were submitted for a ClustalW (http://npsa-prabi.ibcp.fr) multiple sequence alignment. The degree identity and conservation of each residue was then determined using the Jalview Java Alignment Editor [26].

### 3.6. Antigenicity of the N-PfCSP^68–87^-GlyGly-STARP^24–43^-GlyGly-Pf155/RESA^141–160^-GlyGly-MSA1^1283–1301^-GlyGly-C Large Chimera Peptide

Antigenicity studies were performed by immunizing groups of 3–5 weeks of age female BALB/c mice with each peptide component (four single peptides representing *Pf*CSP, STARP, MSA1 and *Pf*155/RESA), the 82-residue chimera, as well as a group was administered with a mixture of the four peptide components and finally an animal group vaccinated with a mixture composed by all single peptide components plus the 82-reside chimera. Besides a placebo group was administered with saline solution. Peptide vaccine formulations were prepared on Freund’s complete adjuvant for the first immunization and with Freund’s incomplete adjuvant for all boosts. Immunization schedule consisted in a first vaccine dose and four boosts at days 14, 21, 28 and 35. Bleeding samples were obtained previously to the first vaccination and after the third, fourth and fifth administrations.

Immunochemistry studies were conducted for assaying reactivity of antibodies anti-each peptide component of the chimera, as well as the chimeric molecule itself. Specific anti-test compound activity was detected using a proper dilution of either anti-mouse or an anti-rabbit IgG-peroxidase-conjugates for Enzyme-Linked Immunosorbent Assay (ELISA) tests and anti-mouse IgG-FITC (fluorescein isothiocyanate) conjugates (Vector Laboratories, Inc., Burlingame, CA, USA) for Indirect Fluorescent Antibody (IFA) tests, which were conducted by analyzing antibody sera reactivity against *Plasmodium*´s blood-stages natively expressed proteins on infected RBC extended on glass slides and simultaneously against sporozoite NF54 *P. falciparum* forms extended on glass slides.

### 3.7. Functional In Vivo Activity of the Chimeric Peptide on Plasmodium berghei and Plasmodium yoelii Infected BALB/c Mice

After antigenicity studies have proven developing high antibody titers in vaccinated animals, each test group of animals was splitted and got prepared for a *Plasmodium* infection by experimental challenging with *P. berghei* and *P. yoelii* strains respectively. As previously published, for experimental challenging, both in vivo obtained strains, showing a 40–50% range of parasitaemia, were independently centrifugated at 1500 rpm for 5 min; the pellet containing about 1 × 10^7^ infected red blood cells (iRBCs) was suspended in 1 mL RPMI and immediately used for either intravenously or intraperitoneal inoculated, each animal was administered with 5 × 10^4^ iRBCs. Parasitemia in all infected animals was monitored by Wright and acridine-orange staining of blood-smear, showing that mice became parasitized by the second to sixth day after infection, and that parasitemia levels increased faster until animals died 12 to 15 days after having been infected. Parasitaemia follow-up was performed accordingly to the experiment requirements. Animal care was in accordance with international guidelines. Animals subjected to invasive procedures were treated under anesthetic, analgesic and tranquilizing and intra muscularly administrated with a combination of 150 mg/kg ketamine/10 mg/kg, xylazine [27,28].

## 4. Conclusions

The present approach for obtaining novel synthetic large immunogens have considered selection and rational positioning into a large construct of four relevant *Plasmodium falciparum* antigens derived from two different living-cycle stages. Selected antigens were each rationally modified by performing strategic amino acid substitutions into their binding motifs and subsequently Gly-Gly spacers were used in order to serve as possible substrate processing substrates on APCs. Obtained results configure important evidence supporting this molecular design.

Altogether the herein presented evidence disclose a new strategy for obtaining large-chimerical peptides useful for presenting multiple antigens form different pathogen’s infective stages and so represents a feasible methodology in vaccine candidate discovery, in spite that the immune mechanisms related to this molecules protecting capacity remain to be elucidated and experienced in animal models closer to humans.

## Figures and Tables

**Table 1:** *Plasmodium falciparum* targets and its large chimera immunogen characteristics.

Protein-Antigen	Peptide/Modified	Amino-acid Sequence	Molecular Weight (Da)	Molecular Mass *m*/*z* [M + H^+^]	Retention Time (min)
*Pf*CSP^68–87^	4383	NSRSLGEN**DDGNN**EDNEKLRY	1487.55	2439.31	17.76
	**25607**	NNSFSLGE**NPNAN**P***GG***	1587.59	1589.32	23.89
	25608	***CG***NNSFSLGE**NPNAN**P***GC***	1809.30	1810.07	18.36
STARP^24–43^	20546	VIKHNRF**LSE**YQSNFLGGGY	2329.90	2629.91	26.56
	**24319**	VIKHMRF**HAD**YQAPFLGGGY***G***	2420.73	2422.15	17.86
	24320	***CG***VIKHMRF**HAD**YQAPFLGGGY***GC***	2628.60	2426.00	24.00
MSA1^1182–1301^	1585	EVLY**LKP**LAGVYRSLKKQLE	2346.79	2348.10	27.94
	**10013**	EVLY**HVP**LAGVYRSLKKQLE***GG***	2455.83	2456.14	27.64
	10014	***CG***EVLY**HVP**LAGVYRSLKKQLE***GC***	2663.50	2662.91	26.63
*Pf*155/RESA^141–160^	6671	MTDVNRYRYSNNYE**AI**PHIS	2442.64	2444.08	20.42
	**9947**	MTDVNRYRYSNNYE**EQ**PHIS***G***	2629.75	2630.50	18.97
	9948	***CG***MTDVNRYRYSNNYE**EQ**PHIS***GC***	2836.04	2836.25	17.90
Chimera^1–82^	39543	KNSFSLGE**NPNAN**P***GG***VIKYH	9072.11	9072.81	19.39 ^a^
		MRF**HAD**YQAPFLGGGY***GG***EVL			26.30 ^b^
		Y**HVP**LAGVYRSLKKQLE***GG***MT			
		DVNRYRYSNNYEE**EQ**HIS***GG***			

Amino acid sequences for each *Plasmodium*’s protein derived native peptide are in the top side of each box and their relevant residues are underlined. Modified sequences in even numbers denote monomer forms employed for structure analyses; their corresponding sequences in odd numbers for polymer forms used for biological studies. ^a^ HPLC-retention time for a 45 min 0–70% acetonitrile linear gradient and ^b^ retention time for a 45 min 21–41% acetonitrile linear gradient respectively.

**Table 2:** Experimental design for immunogen formulation and animal immunization.

Group	Mouse	Immunogen-Code	Origin-Antigen	Parasite Stage-Code
1	1–4	9948	*Pf*155/RESA^141–160^	MZ1
2	5–8	10014	MSA1-^1182–1301^	MZ2
3	9–12	24320	STARP^24–43^	SPZ1
4	13–16	25608	*Pf*CSP^68–87^	SPZ2
5	17–20	Chimera	All consecuti	SPZ-1-SPZ2-MZ1-MZ2
6	21–24	M1	SPZ1 + SPZ2 + MZ1 + MZ2	Mixture **1** (**M1**)
7	25–28	M2	[SPZ1 + SPZ2 + MZ1 + MZ2] + Chimera	Mixture **2** (**M2**)
8	51–54	Placebo	Saline solution	Placebo

Conventions used for the chimera construct: SPZ1-SPZ2-MZ1-MZ2 which is equivalent to fragment peptides organization being: *Pf*CSP-STARP-MSA1-*Pf*155/RESA. SPZ for sporozoite and MZ for merozoite components.

## Supplementary Materials

The following are available online.
