# Supplementary material for: A Large Size Chimeric Highly Immunogenic Peptide Presents Multistage Plasmodium Antigens as a Vaccine Candidate System against Malaria
Source: Molecules. 2017 Nov 1;22(11):1837. doi: 10.3390/molecules22111837 (PMC6150380; doi:10.3390/molecules22111837)
Supplement: Supplementary file 1 [file molecules-22-01837-s001.pdf]

Supplementary material and Figure S1

Molecular identity and physicochemical features

Purity of all products was ensured by semi-preparative RP-HPLC to higher than 95%. **Figure S1A** and **C** in Supplementary material displayed chromatographic profiles for the large chimeric 82-amino-acid peptide obtained by standard and microwave-assistance respectively. As observed, retention times were 26.38 min and 26.30 min respectively evidencing identical hydrophobic properties regarding each other. Besides, MALDI-TOF mass spectrometry allowed identifying molecular [M+H+] ions at 9072.806 amu and 9076.460 amu respectively concerning the expected 9072.11 Da molecular weight as seen in **Figure S1A** and **C** in Supplementary material.

Purified molecules were submitted to CD analyses as described in the Methods and material for determining predominant secondary structure elements in solution of all molecules. Therefore CD assays were performed in two different hydrophobicity systems, water and 30% trifluoroethanol-water. As observed in **Figure S1E** in Supplementary material, most molecules analyzed in water revealed random coiled molecular arrangements, while the *Pf*155/RESA (peptide **9948**, blue profile) resembles a turn-like conformation and the MSA1 (peptide **10014**, orange profile) show a weak disposition to an α-helix conformation preference. On the other hand the whole molecule family was analyzed in the second system being highly hydrophobic, thus STARP (**24320** green line) and MSA1 (**10014** orange line) analogues evidenced strong α-helix contents as judged by minimal ellipticity values at 208 nm and 222 nm and a maximum at 190 nm as the predominant secondary structure element as observed in **Figure S1F** in Supplementary

material. Meanwhile *Pf*155/RESA (**9948** blue line) and *Pf*CSP (**25608** ochre line) analogues agreed to more relaxed random-coiled conformations. Interestingly, when a mixture composed by all four single peptide components was analyzed by CD experiments, its secondary structure pattern resembles a strong α-helix (pink line). Hence secondary structural features revealed by the peptide mixture are mainly governed by those highly organized α-helices present in two components of the mixture one belonging to erythrocyte stage and to the pre-erythrocyte stage the other, it seemed that both of them strongly influenced the solution structure global behavior. The large-chimera peptide (**39543**) revealed secondary structure elements resembling α-helical conformations as described by light-blue profile in the figure.

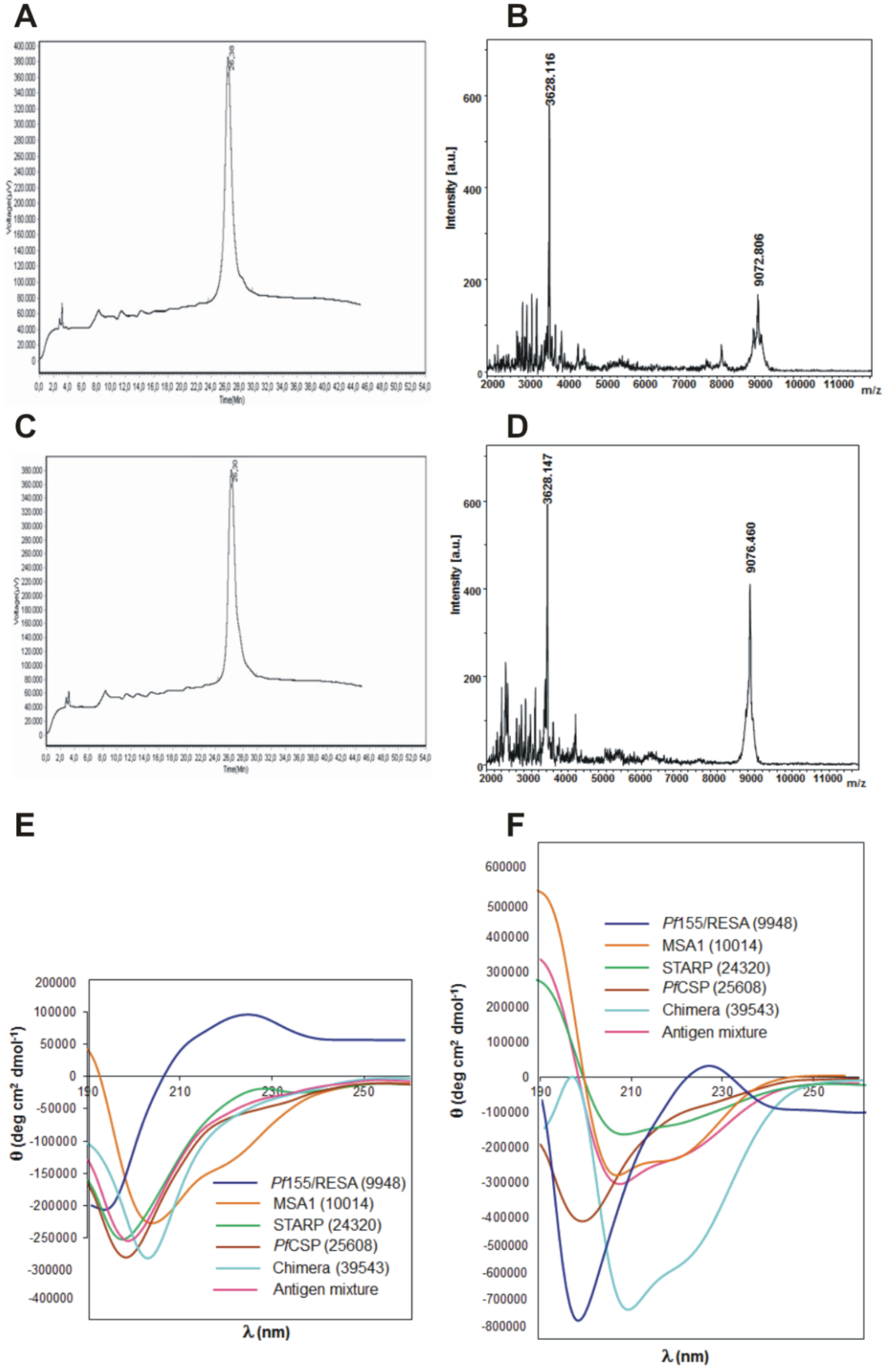

Figure 1S. Physicochemical identity of produced large-chimeric peptide and secondary structure elements in all single antigen components and the 82-large chimeric immunogen. A. RP-HPLC pattern for the 82-residue chimera obtained by

standard solid phase peptide synthesis strategies. **B**. Mass spectrometry identification of the standard solid phase obtained 82-chimera peptide. **C**. RP-HPLC pattern for the microwave assisted solid phase synthesis 82-chimera peptide. **D**. Mass spectrometry identification of the microwave assisted solid phase synthesis 82-chimera peptide. Solvent system linear gradient was conducted from 0- 70 %B over 45 minutes (Mobile phase A: H_2O/0.05% trifluoroacetic acid (TFA), B: acetonitrile (ACN)/0.05% TFA). Injection samples of 80 μL volume of peptide solutions of 1 mg/mL dissolved in H_2O/ACN (1:1) were analyzed. Relative ion abundancy is shown (y axis) and the molecular mass for formed ions is shown as atomic mass units (amu) on the m/z (x axis). CD experiments for all molecule systems were analysed in water **E.** and 30% TFE-water in **F.** Results are expressed as mean residue ellipticity [θ], the units being degrees x cm^2 x $dmol^{-1}$ accordingly to the $[\theta] = \theta_\lambda/(100lcn)$ function, where θ_λ was measured ellipticity, *l* the optical path length, *c* molecule concentration and *n* the number of amino acid residues in the sequence.
